# Cholera Toxin Promotes Th17 Cell Differentiation by Modulating Expression of Polarizing Cytokines and the Antigen-Presenting Potential of Dendritic Cells

**DOI:** 10.1371/journal.pone.0157015

**Published:** 2016-06-06

**Authors:** Jung-Ok Kang, Jee-Boong Lee, Jun Chang

**Affiliations:** 1 Graduate School of Pharmaceutical Sciences, Ewha Womans University, Seoul, Korea; 2 Department of Molecular Cell Biology, Sungkyunkwan University School of Medicine, Suwon, Kyunggi-Do, Korea; Seoul National University College of Pharmacy, REPUBLIC OF KOREA

## Abstract

Cholera toxin (CT), an exotoxin produced by *Vibrio cholera*, acts as a mucosal adjuvant. In a previous study, we showed that CT skews differentiation of CD4 T cells to IL-17-producing Th17 cells. Here, we found that intranasal administration of CT induced migration of migratory dendritic cell (DC) populations, CD103^+^ DCs and CD11b^hi^ DCs, to the lung draining mediastinal lymph nodes (medLN). Among those DC subsets, CD11b^hi^ DCs that were relatively immature had a major role in Th17 cell differentiation after administration of CT. CT-treated BMDCs showed reduced expression of MHC class II and CD86, similar to CD11b^hi^ DCs in medLN, and these BMDCs promoted Th17 cell differentiation more potently than other BMDCs expressing higher levels of MHC class II and CD86. By analyzing the expression of activation markers such as CD25 and CD69, proliferation and IL-2 production, we determined that CT-treated BMDCs showed diminished antigen-presenting potential to CD4^+^ T cells compared with normal BMDCs. We also found that CT-stimulated BMDCs promote activin A expression as well as IL-6 and IL-1β, and activin A had a synergic role with TGF-β1 in CT-mediated Th17 cell differentiation. Taken together, our results suggest that CT-stimulated DCs promote Th17 cell differentiation by not only modulating antigen-presenting potential but also inducing Th polarizing cytokines.

## Introduction

Dendritic cells (DCs) are centrally involved in initiation of adaptive immunity and are activated by ligation of PRRs (pattern recognition receptors) with exogenous PAMPs (pathogen-associated molecular patterns) such as lipopolysaccharides (LPS), CpG or poly(I:C) [1]. DCs are professional antigen-presenting cells that present antigens through the MHC molecules to T cells, stimulating their activity. DCs consist of plasmacytoid DCs (pDCs), conventional DCs (cDCs) and migratory DCs (mDCs). Of those DC subsets, mDCs have an important role in protecting the residing tissue through sampling antigens that enter from outside and transferring them to T cells in the draining lymph nodes. DCs stimulate T cells by antigenic signaling, costimulatory signaling and dictating Th polarization through cytokine production. Following stimulation with adjuvants such as PAMPs, DCs increase expression of costimulatory molecules or produce IL-12, IL-4 or IL-6 and TGF-β which are Th1-, Th2- and Th17-polarizing cytokines, respectively. Therefore, adjuvants have a critical role in Th differentiation by stimulating DCs to produce polarizing cytokines.

Emerging evidence suggests that the quantity and quality of TCR signaling also have a critical role in addition to the polarizing cytokine milieu [2–7]. Based on *in vitro* and *in vivo* studies, it has been suggested that signaling through the TCR and costimulatory receptors can dictate the polarization of Th development. Development of Th1 cells is favored by a high dose of peptide or a strongly agonistic ligand whereas Th2 cell development is favored by a low dose of peptide or a weakly agonistic ligand. It has also been shown that Th cell development is dictated by the interplay of antigen and cytokine signals. Under each polarizing condition, Th1 cells are efficiently generated by a high dose antigen, Th17 cells by an intermediate dose antigen, and Th2 cells by a low dose antigen [6]. In addition to the antigen concentration, adjuvants can also influence Th polarization *in vivo* by modulating TCR-dependent signal intensity [7].

In a previous study, we showed that the mucosal adjuvant cholera toxin (CT), which is an exotoxin produced by *Vibrio cholera*, promotes Th17 differentiation to bystander antigens [8]. Other studies suggested that CT stimulates DC to produce IL-6 and IL-1β, which play critical roles in Th17 differentiation [9]. However, it is unknown which DC subsets and mediators are involved in Th17 differentiation driven by CT administration. To this end, we studied the role of DCs and associated cytokines in Th17 differentiation by CT treatment through DC-T cell co-culture, DC-free T cell stimulation and *in vivo* immunization study. We show here that intranasally administered CT induced migration of migratory DC populations, CD103^+^ DCs and CD11b^hi^ DCs, to the lung draining lymph nodes. CD11b^hi^ DCs are more important in Th17 differentiation than CD103^+^ DCs, which migrated extensively to the lung draining lymph node and showed a more mature phenotype. Moreover, we found that CT-stimulated BMDCs produce activin A, which is a member of the TGF-β family, and neutralization of activin A significantly decreased Th17 differentiation by CT-stimulated BMDCs. We also found that the ability of CT-treated BMDCs to direct Th17 differentiation was significantly decreased under a high-dose antigen condition. In addition, CT treatment increases low expressers of MHC class II and CD86 in the BMDC population, which promotes more extensive Th17 cell differentiation than high expressers of MHC class II and CD86, suggesting that CT can direct Th cell differentiation by controlling the antigen-presenting potential in DCs. Together, these data suggest that CT promotes Th17 cell differentiation by not only inducing polarizing cytokines but also modulating antigen-presenting potential.

## Materials and Methods

### Mice and ethics statement

Female C57BL/6 (B6) mice and BALB/c mice were purchased from Orient Bio (Seoul, Korea). OT-II TCR transgenic mice and IL-6 KO mice (B6 background), were from the Jackson Laboratory (Bar Harbor, ME). Mice were maintained under specific pathogen-free condition and were used between 6 and 10 weeks of age. All animals were handled in strict accordance with good animal practice as defined by the relevant national and/or local animal welfare bodies, and all animal work was approved by Ewha Womans University’s institutional animal care and use committee (IACUC, Approval Number.15-069).

### Reagents

CT was purchased from List Biological Laboratories (Campbell, CA). GM1 ganglioside was purchased from Calbiochem (La Jolla, CA). Peptides were synthesized from Peptron Inc. (Daejon, Korea). Antibodies for flow cytometric analysis were from BioLegend (San Diego, CA) or BD Bioscience (San Diego, CA). Neutralizing antibodies were purchased from eBioscience (San Diego, CA) or R&D (Minneapolis, MN). LPS, PMA, ionomycin, SB431542 and SB203580 were purchased from Sigma-Aldrich (St. Louis, MO).

### Generation of BMDCs

Bone marrow derived dendritic cells (BMDCs) were generated from bone marrow of B6 or *IL-6*^*-/-*^ mice by culturing in complete RPMI medium containing 10% FBS and 50 μM 2-mercaptoethanol supplemented with 10 ng/ml recombinant GM-CSF and IL-4 (R&D Systems). The bone marrow was obtained from mice euthanized by carbon dioxide (CO_2_) inhalation. After 7 days of culture, non-adherent cells were harvested by gentle pipetting, and BMDCs were enriched for CD11c^+^ cells by using CD11c MicroBeads (Miltenyi Biotec).

### Analysis of lung migratory dendritic cells and BMDCs

Mice (n = 15) were i.n. administered with 2 μg of CT and medLN cells were prepared before or 1–3 days after the administration. For i.n. administration, mice were lightly anesthetized by isoflurane (Ifran®, Hana Pharm, Kyounggi-Do, Korea) inhalation and CT in a volume of 50 μl of phosphate-buffered saline (PBS) was applied to the left nostril. The CT-administered mice didn’t have any pathologic appearance compared to untreated mice during the days. MedLNs were removed from the mice euthanized by CO_2_ inhalation and passed through a 70 μm mesh cell strainer to obtain single cells. The DC phenotype was determined after staining with fluorescein isothiocyanate (FITC)-conjugated MHC II (M5/114.15.2; BioLegend), peridinin-chlorophyll-cyanin5.5 (PerCPCy5.5)-conjugated CD11c (N148; eBioscience), phycoerythrin (PE)-conjugated CD11b (M1/70; eBioscience), and allophycocyanin (APC)-conjugated CD103 (2E7; eBioscience). Flow cytometry was conducted on a FACSCalibur (BD) and analyzed with FlowJo software (TreeStar).

For analyzing maturation status of DCs, medLN cells were prepared 2 days after administration with PBS or 2 μg of CT and stained with FITC-conjugated MHC II (M5/114.15.2; BioLegend), PerCPCy5.5-conjugated CD11c (N148; eBioscience), allophycocyanin-e780-conjugated CD11b (M1/70; eBioscience), APC-conjugated CD103 (2E7; eBioscience), and PE-conjugated CD40 (1C10; eBioscience), CD80 (16-10A1; BioLegend), and CD86 (GL1; eBioscience). Flow cytometry was conducted on an LSRII (BD) and analyzed with FlowJo software (TreeStar).

For analyzing maturation status of CT-treated BMDCs, BMDCs were treated with CT (2 μg/ml) for a day and stained with FITC-conjugated MHC II (M5/114.15.2; BioLegend), PerCPCy5.5-conjugated CD11c (N148; eBioscience), and PE-conjugated CD40 (1C10; eBioscience), CD80 (16-10A1; BioLegend), and CD86 (GL1; eBioscience). Flow cytometry was conducted on a FACSCalibur (BD) and analyzed with FlowJo software (TreeStar).

### Real time PCR

Total RNA was isolated using QIAzol lysis reagent according to manufacturer’s instructions (Qiagene). cDNA was synthesized with the RT-&GO Mastermix (MP Biomedicals, CA), and real-time PCR was performed on a Bio-Rad CFX 96 Touch Real-Time PCR Detection System with Power SYBR Green PCR Mastermix (AB Systems) and primer pairs specific for cDNA of *nodal*, *activin* a, *activin bA*, *activin bB*, *tgf-b1*, *tgf-b2*, *tgf-b3*, *Il6*, *Il1b*, *gapdh*, *and bactin* transcripts. The primer sets were as follows: *nodal*, 5’-TGGCGTACATGTTGAGCCTCT-3’ (forward) and 5’-TGAAAGTCCAGTTCTGTCCGG-3’ (reverse); *activin a*, 5’-ATGTATTCCGGCCATCCCAA-3’ (forward) and 5’-CTACCATGGCAGTAGTGGAA-3’ (reverse); *activin bA*, 5’-GAGAGGAGTGAACTGTTGCT-3’ (forward) and 5’-TACAGCATGGACATGGGTCT-3’ (reverse); *activin bB*, 5’-GTTCTTCATCGACTTTCG-3’ (forward) and 5’-CGATCATGTTGGGCAC-3’ (reverse); *tgf-b1*, 5’-GCTGCGCTTGCAGAGATTAAA-3’ (forward) and 5’-TTGCTGTACTGTGTGTCCAG-3’ (reverse); *tgf-b2*, 5’-CCAAAGACTTAACATCTCCCACC-3’ (forward) and 5’-GTTCGATCTTGGGCGTATTTC-3’ (reverse); *tgf-b3*, 5’-GCTCTTCCAGATACTTCGAC-3’ (forward) and 5’-AGCAGTTCTCCTCCAGGTTG-3’ (reverse); *Il6*, 5’-TCCAGTTGCCTTCTTGGGAC-3’ (forward) and 5’-GTACTCCAGAAGACCAGAGG-3’ (reverse); *Il1b*, 5’-CCAGCTTCAAATCTCACAGCAG-3’ (forward) and 5’-CTTCTTTGGGTATTGCTTGGGATC-3’ (reverse); *gapdh*; 5’-AATGTGTCCGTCGTGGATCT-3’ (forward) and 5’-TCCACCACCCTGTTGCTGTA-3’ (reverse); *bactin*, 5’-CCGGGACCTGACAGACTA-3’ (forward) and 5’-GTTTCATGGATGCCACAGGAT-3’ (reverse). Reactions were run in triplicate and samples were normalized to *gapdh* and *bactin* as induction relative to expression in control samples. Primers for *bactin* and *gapdh* were designed using Primer3-Blast (NCBI). Primers for *Il6* and *Il1b* were as published [10]. Except primers for *bactin*, *gapdh*, *Il6* and *Il1b*, all other primers were as described [11].

### Cytokine assays

BMDCs were incubated with PBS or CT (2 μg/ml) for the indicated days and the culture supernatants were harvested at the indicated time points. The levels of specific cytokines were determined by ELISA kit for TGF-β1 (R&D Systems) and cytokine bead assay for MCP-1, MCP-3, MIP-1α, MIP-1β, GM-CSF, TNF-α, IL-1β, IL-6, IL-10, IL-18, and IL-23 (eBioscience).

OT-II CD4^+^ T cells were incubated with untreated or CT-treated BMDCs pulsed with variable concentration of OVA_323-339_ peptide. At day 5, the culture supernatant was removed to measure the level of IL-2 by cytokine bead assay (eBioscience).

### *In vitro* T cell differentiation

To induce T cell differentiation in DC-assisted system, isolated in vivo migratory DC subsets, BMDCs (untreated or CT-treated), or phenotype-based, isolated BMDCs were used. *In vivo* DCs were isolated from medLN cells of mice 2 day after i.n. CT administration. MedLN cells were first enriched for CD11c^+^ cells using CD11c MicroBeads (Miltenyi Biotec) and CD11c^+^CD8α^-^CD11b^hi^CD103^-^ DCs (CD11b^hi^ DC) and CD11c^+^CD8α^-^CD11b^-/lo^CD103^+^ DCs (CD103^+^ DC) were FACS sorted using FACSAria II (BD Bioscience).

The BMDCs were incubated with CT for a day and in some experiments, CT-treated BMDCs were FACS sorted using FACSAria II (BD Bioscience) based on CD86 expression. The isolated DC subsets or BMDCs were pulsed with indicated concentration of OVA_323-339_ peptide for 1 h and washed sufficiently before culturing with CD4^+^ T cells. Isolated CD4^+^ T cells (Miltenyi Biotec) from OT-II mouse spleens were then incubated with the DCs. After 5 days, the T cells were restimulated with PMA (50 ng/ml) and ionomycin (500 ng/ml) in the presence of Brefeldin A (BD Biosciences). After 5 h, the cells were then analyzed for cytokine production.

To induce T cell differentiation in a DC-free system, naïve CD4^+^ T cells isolated using CD4^+^ T Cell Isolation kit (Miltenyi Biotec) from spleens of OT-II mice were stimulated for 5 days with plate-bound anti-CD3 (2 μg/ml; 145-2C11, BioLegend), soluble anti-CD28 (1 μg/ml; 37.51, BD Bioscience), anti-IFN-γ (10 μg/ml; XMG1.2, BioLegend) in the absence or presence of 10% BMDC conditioned media (CM) or CT-BMDC CM. BMDC CMs were prepared from BMDC cultures untreated or treated with 2 μg/ml of CT for 2 days and CT in BMDC CM was blocked by pre-incubating with 10-fold molar excess of GM1 ganglioside before being added to the culture. Other neutralizing antibodies were used to block a specific cytokine in the BMDC CM: anti-IL-1β (10 μg/ml; B122, eBioscience); anti-TGF-β1 (20 μg/ml; 1D11.16.8, eBioscience); anti-activin A (20 μg/ml; 69403, R&D Systems). SB431542 and SB203580 were added to the culture in 10 μM of concentration. After 5 days, the cells were then restimulated with phorbol myristate acetate (50 ng/ml) and ionomycin (500 ng/ml) to assess cytokine production in the presence of Brefeldin A for 5 h.

### Surface staining, intracellular cytokine staining, and flow cytometric analysis

For flow cytometry and intracellular cytokine staining, the following mAbs were used: FITC-conjugated anti-CD3 (17A2; BioLegend); phycoerythrin-cyanin 5-conjugated anti-CD4 (RM4-5; BD Bioscience); PE-conjugated anti-IL-17A (TC11-18H10.1; BioLegend); APC-conjugated anti-IFN-γ (XMG1.2; BioLegend); FITC-conjugated anti-CD25 (PC61; BioLegend); PE-conjugated anti-CD69 (H1.2F3; BioLegend); PE- or APC-conjugated anti-Vα2 TCR (B20.1; BD Biosciences). Samples were acquired on FACSCalibur (BD Biosciences) or LSRFortessa (BD Biosciences), and data were analyzed with FlowJo software (TreeStar).

### Measurement of CD25 and CD69 following activation

8x10^4^ OT-II CD4^+^ T cells were co-cultured with 2x10^4^ BMDCs antigen-pulsed in graded concentration of OVA_323-339_ peptide. After 16 h, OT-II CD4^+^ T cells were harvested and determined for CD25 and CD69 in CD4^+^Vα2 TCR^+^ T cells.

### Cell proliferation assay

OT-II CD4^+^ T cells (1x10^7^) were labeled in 1 ml of PBS containing 5 μM CFSE (eBioscience) for 15 min at room temperature. 8x10^4^ CFSE labeled OT-II CD4^+^ T cells were cultured with 2x10^4^ BMDCs pulsed with OVA_323-339_ peptide in graded concentration of OVA_323-339_ peptide. At 96 h, OT-II CD4^+^ T cells stimulated under each condition were harvested and determined for CFSE in CD4^+^Vα2 TCR^+^ T cells.

### Statistical analysis

Two-tailed Student’s t test was used for comparison of means, and values of P<0.05 were considered statistically significant.

## Results

### CD11b^hi^ DCs and CD103^+^ DCs migrate to lung draining lymph nodes after CT administration, and CD11b^hi^ DCs contribute to Th17 differentiation

**Fig 1:**
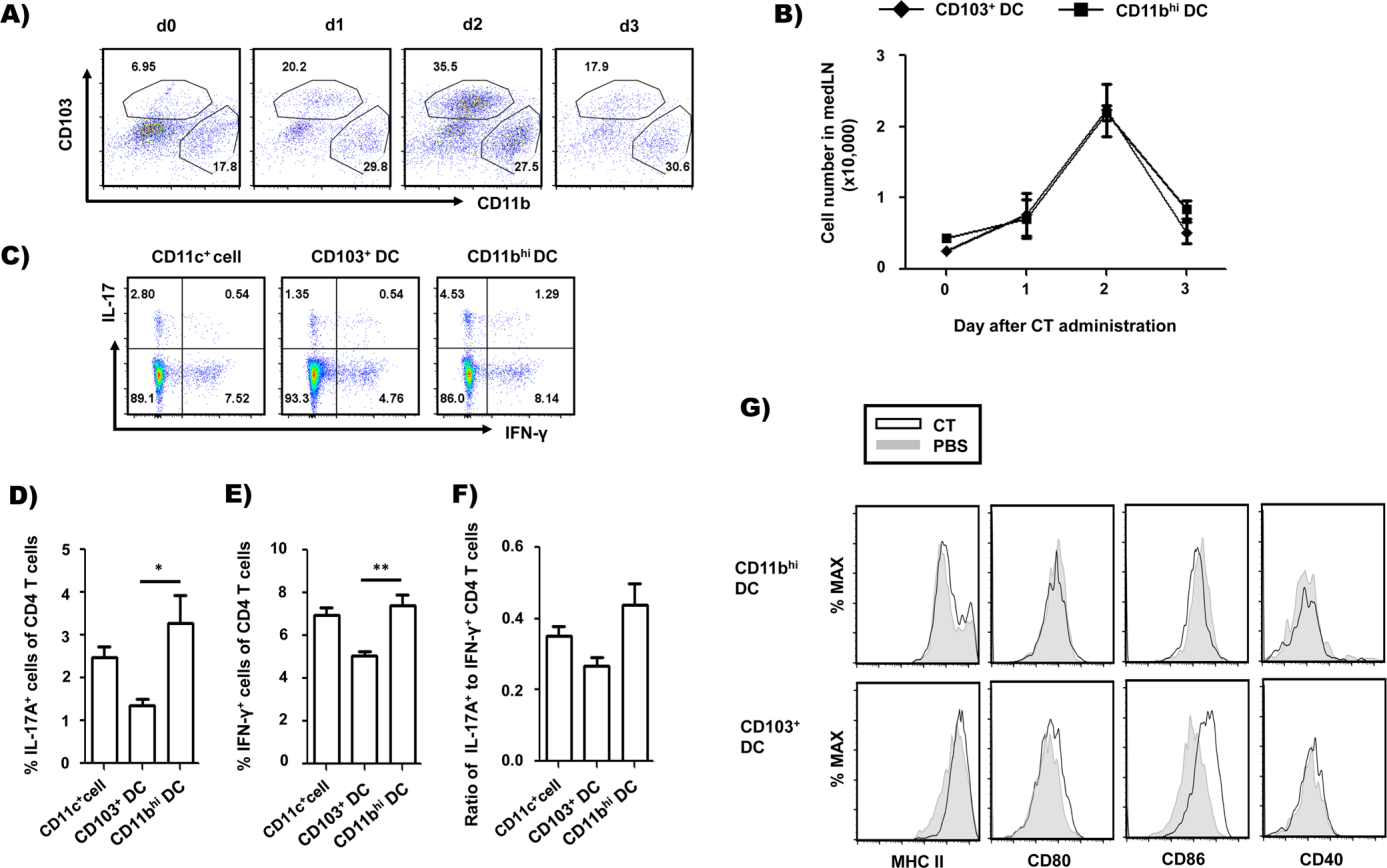
CD11b^hi^ DCs and CD103^+^ DCs migrated to the lung draining lymph node after CT administration and CD11b^hi^ DCs with the MHCII^lo^CD86^lo^ phenotype mediated IL-17-favored differentiation of CD4 T cell. Mice were intranasally administered 2 μg CT, and mediastinal lymph node (medLN) cells were prepared from the mice at day 1, day 2, or day 3 after or before CT administration. (A) Flow cytometry analysis of migratory DC subsets after CT administration. Five mice per group; migratory DC subsets in medLN (CD103^+^ DC and CD11b^hi^ DC); (B) Cell number of each DC subset after CT administration; (C-F) CD103^+^ DCs and CD11b^hi^ DCs were isolated from medLN cells of mice 2 day after intranasal CT administration, pulsed with 0.1 μM of OVA_323-339_ peptide and washed and cultured with OT-II CD4^+^ T cells for 5 days. Stimulated OT-II CD4^+^ T cells were analyzed for expression of IL-17A and IFN-γ by flow cytometry after restimulation with PMA and ionomycin. (C) Flow cytometry analysis and (D and E) frequency of IL-17A- and IFN-γ-expressing CD4^+^ T cells. (F) Ratio of IL-17A^+^ to IFN-γ^+^ CD4^+^ T cells. (G) MHC class II and costimulatory molecules on DC subsets isolated from medLN of mice 2 day after administration of CT or PBS. *p<0.05, **p<0.01 (Student’s *t*-test). Data are from one experiment representative of two independent experiments with similar results. Dot plots and histograms are representative of four mice in A and G and quadruplicate wells in C and data are average ± SEM.

To identify which DC subsets have a critical role in Th17 cell differentiation by CT *in vivo*, we analyzed DC subsets that migrated to the draining lymph node after intranasal CT administration. Mediastinal lymph nodes (medLN), which are lung draining lymph nodes, were prepared daily for 3 days after administering CT intranasally, and the medLN cells were analyzed for migratory DC subsets (Fig 1A and 1B). We identified migratory DC subsets on the basis of expression of CD11c, MHC II, CD103 and CD11b, as suggested by the previous reports [12–14]. One major subset was the CD11c^+^MHCII^+^CD103^+^CD11b^lo/-^ (CD103^+^ DC) group and the other was the CD11c^+^MHCII^+^CD103^-^CD11b^hi^ (CD11b^hi^ DC) group. CD103^+^ DCs and CD11b^hi^ DCs in the draining lymph nodes were analyzed by flow cytometry. As shown in Fig 1, both DC subtypes were recruited in the draining lymph nodes after CT administration, and the migration of DCs peaked at day 2. However, both DC subsets were diminished on day 3 after CT administration.

To determine which DC subset primarily mediates Th17 differentiation by CT administration, we assessed the capability of each CD11b^hi^ DC and CD103^+^ DC subset to differentiate Th17 cells. Each DC subset was isolated from the medLN at day 2 after intranasal CT treatment and was co-cultured with naïve CD4 T cells. Compared to CD103^+^ DCs, CD11b^hi^ DCs were able to promote more differentiation of IL-17-producing T cells including IFN-γ-producing T cells (Fig 1C–1F). These results suggest that CD11b^hi^ DCs have an important role as a cellular mediator in Th17 differentiation *in vivo* following intranasal CT administration. To investigate phenotypic changes between the two DC subsets, we compared the expression of MHC class II molecules and costimulatory molecules such as CD80 and CD86 in each DC subset. As shown in Fig 1G, the levels of MHC class II, CD80, and CD86 were increased in CD103^+^ DCs from CT-treated mice compared to those from the control PBS mice. In contrast, there was no significant change in the expression of these markers in the CD11b^hi^ population. These results show that CD103^+^ DCs migrate to the medLN as relatively mature upon CT administration, while CD11b^hi^ DCs migrate while sustaining an immature state. Nonetheless it seems that CD11b^hi^ DCs have higher CD80 and CD86 on intensity compared to CD103^+^ DCs, contrasting with relatively reduced MHC class II molecule.

### CT increases a population of BMDCs showing reduced expression of class II MHC and CD86 that mediates Th17 differentiation

**Fig 2:**
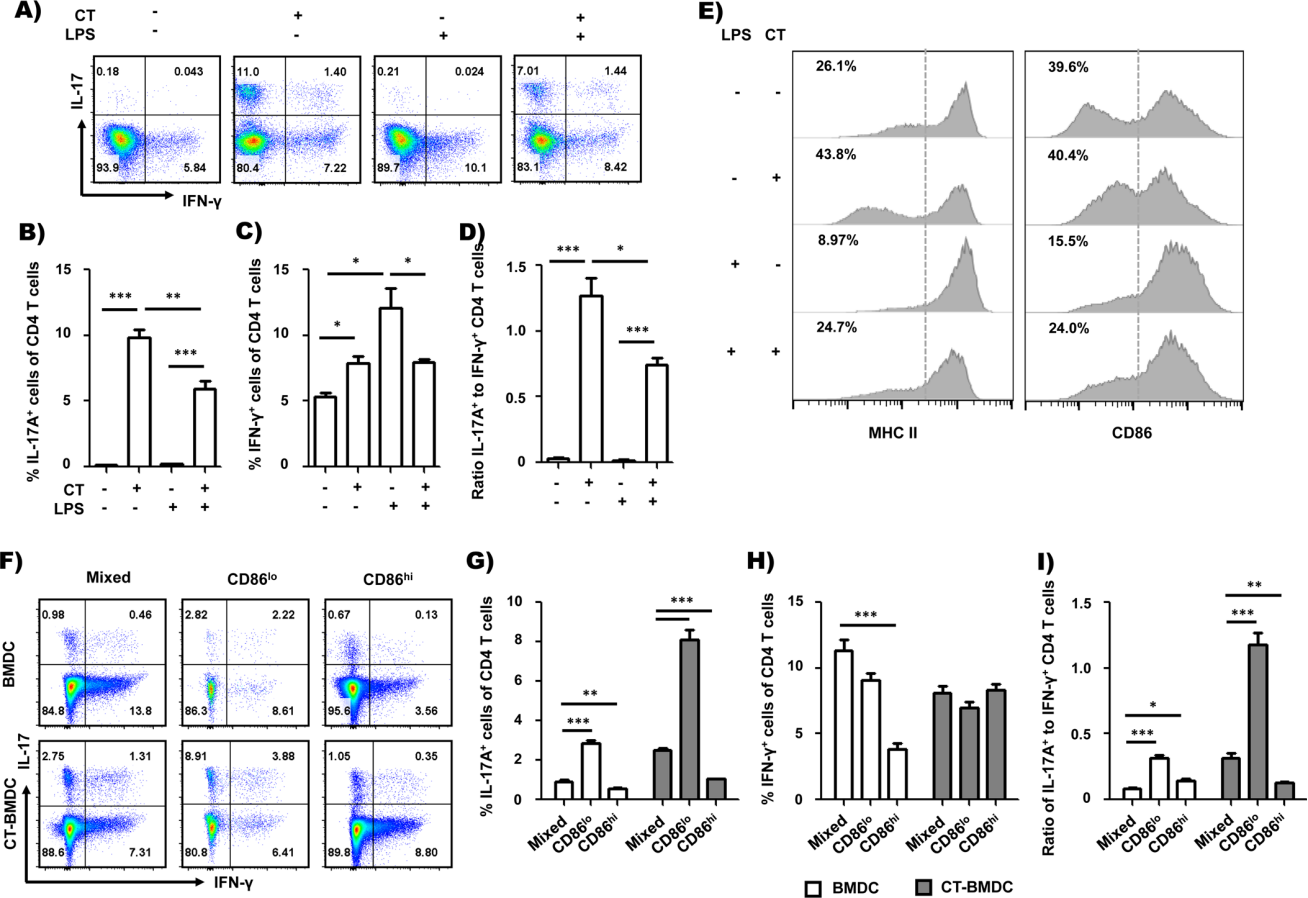
DCs with the MHCII^lo^CD86^lo^ phenotype mediated IL-17-favored differentiation of CD4 T cell. (A-D) OT-II CD4^+^ T cell differentiation by untreated BMDCs or BDMCs treated with CT, LPS or CT and LPS. The BMDCs were pulsed with 1 μM of OVA_323-339_ peptide before co-culture with OT-II CD4^+^ T cells. Frequency of IFN-γ- or IL-17A-expressing CD4^+^ T cells (A-C) and ratio of IL-17A-expressing cells to IFN-γ-expressing cells (D). (E) Surface expression of MHC class II and costimulatory molecules between untreated BMDCs and CT-treated BMDCS. BMDCs were treated with CT (2 μg/ml), LPS (100 ng/ml) or CT and LPS or not treated for 24 hr. (F-I) Comparison of OT-II CD4^+^ T cell differentiation by isolated BMDCs or CT-treated BMDCs based on MHC class II and CD86 expression. Frequency of IFN-γ- or IL-17A-expressing CD4^+^ T cells (F-H) and ratio of IL-17A-expressing cells to IFN-γ-expressing cells (I). *p<0.05, **p<0.01, ***p<0.001 (Student’s *t*-test). Dot plots are the representative of at least two independent experiments and data are average ± SEM of triplicate wells in B-D or quadruplicate wells in G-I.

Recent studies suggest that the quality and quantity of TCR signaling could modulate CD4 T cell differentiation in addition to the cytokine milieu [2–6], and modulating strength of the TCR signal is one of the mechanisms in adjuvant-mediated Th cell differentiation [7]. We determined that CD11b^hi^ DCs, a migratory DC subset migrating to the medLN following CT administration, are important cell mediators in Th17 cell differentiation *in vivo*, but they have immature phenotypes. Accordingly, we decided to investigate whether CT is capable of influencing CD4 T cell differentiation by modulating TCR signaling. We first tested whether CT-treated BMDCs (CT-BMDCs) have the capability to regulate Th17-favored differentiation *in vitro* as CD11b^hi^ DCs do *in vivo*. We analyzed *in vitro* Th cell differentiation in a co-culture model of BMDCs and naïve OT-II CD4^+^ T cells. CT-BMDCs significantly increased Th17 cell differentiation compared to BMDCs (Fig 2A). LPS is an adjuvant that is known to mediate Th1-favored differentiation [15–17]. To further analyze the effect of CT treatment on Th differentiation, we examined whether CT treatment redirects LPS-mediated Th1 differentiation to Th17 cell differentiation. As expected, LPS-BMDCs induced polarized Th1 cell differentiation. However, surprisingly, CT-treated LPS-BMDCs dramatically increased Th17 cell differentiation while reducing Th1 cell differentiation (Fig 2A–2D). This result shows that CT directs BMDCs to induce Th17-skewed differentiation, and this occurs even in an LPS-mediated polarized Th1 cell differentiation condition. Next, we tested whether CT influences the surface expression of MHC class II and costimulatory molecules in BMDCs. We observed that BMDCs are consisted of two populations, one with reduced levels of MHC class II and CD86 molecules (boxed area) and the other with increased levels of MHC class II and CD86 molecules (S1A Fig). Fig 2E and S1A Fig show the increase in a population with reduced levels of MHC class II and CD86 molecules on BMDCs 24 h after CT treatment compared to control BMDCs. And those population displayed different phenotypes. As shown in S1A–S1C Fig, MHC II^lo^CD86^lo^ population of CT-BMDCs displayed decreased intensity of MHC class II but increased intensity of CD86 compared to that of BMDCs. However, the expression of CD40 and CD80 was not altered after CT treatment (data not shown). We analyzed whether CT is also capable of regulating the enhanced expression of MHC class II and CD86 by LPS treatment [15–17]. CT treatment increased a population with low levels of MHC class II and CD86 in LPS-treated BMDCs, which was caused by reduced surface expression both of MHC class II and CD86 (Fig 2E and S1A Fig). And, in the population, intensity of MHC class II and CD86 was decreased and increased, respectively, as in the CT-BMDC CD86^lo^ population (S1B and S1C Fig). These results suggest that CT-induced phenotypic change of BMDCs is associated with Th17-skewed differentiation. To investigate directly whether these phenotypic changes on surface expression of MHC class II and CD86 is associated with the Th17-skewed differentiation, we sorted CT-treated BMDCs on the basis of expression levels of these two molecules. Because BMDCs expressing high levels of CD86 also express high levels of MHC class II, we sorted CT-treated BMDCs on the basis of expression level of CD86 to keep MHC II molecules untouched. After pulsing with OVA peptide, the sorted CT-BMDCs (CD86^hi^ or CD86^lo^) were cultured with OT-II CD4^+^ T cells for 5 days and Th cell differentiation was analyzed by staining for IL-17 and IFN-γ. BMDCs with lower levels of MHC class II and CD86 (CD86^lo^ CT-BMDC) showed significantly enhanced Th17 cell differentiation but not IFN-γ-producing cells compared to those with higher levels of MHC class II and CD86 (CD86^hi^ CT-BMDC) or unsorted CT-BMDCs (Fig 2F–2I). We also tested whether CD86^lo^ BMDC population is capable of promoting more Th17 cell differentiation than CD86^hi^ BMDC population. As shown in Fig 2F–2I, CD86^lo^ BMDC population induced higher frequency of Th17 cells than CD86^hi^ counterpart. These results show that MHC II^lo^CD86^lo^ BMDC populations have enhanced potential in Th17 cell differentiation, irrespective of CT treatment and suggest that CT contributes to polarized Th17 cell differentiation by increasing MHC II^lo^CD86^lo^ population and possibly modulating the TCR signaling strength. It is also interesting to note that MHC II^lo^CD86^lo^ BMDC populations induced increased frequency of IL-17^+^ CD4 T cells but in reduced numbers compared to mixed or MHC II^hi^CD86^hi^ populations (S2 Fig).

### CT reduces TCR signaling strength by decreasing the antigen-presenting potential of BMDCs

**Fig 3:**
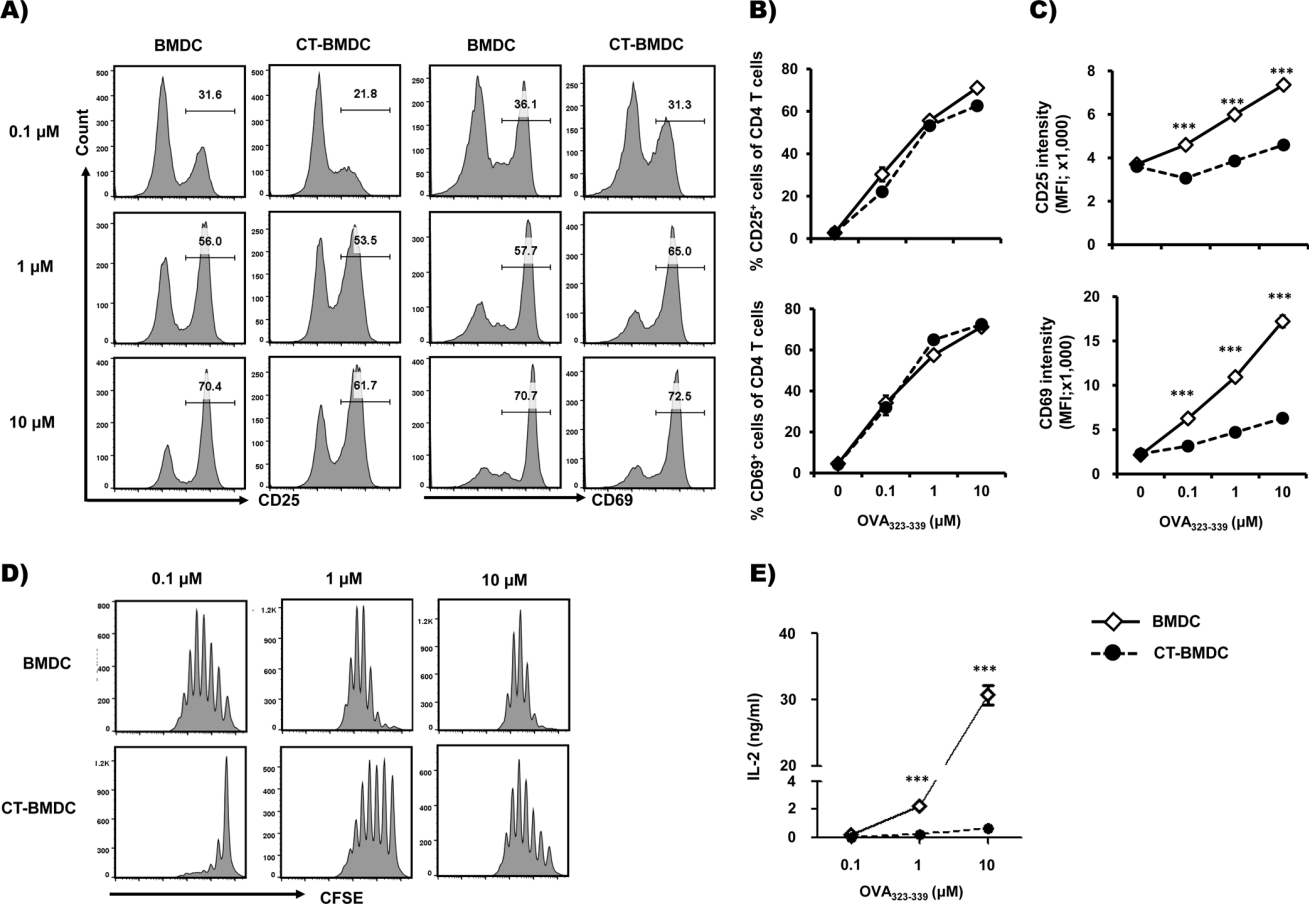
CT-treated BMDCs downregulate T cell activation and IL-2 production. OT-II CD4^+^ T cells were co-cultured with BMDCs or CT-BMDCs pulsed with the indicated concentration of OVA_323-339_ peptide. At 16 h, OT-II CD4^+^ T cells were analyzed for expression of CD25 and CD69 (A-C). Histogram of CD25 and CD69 (A); Frequency of CD25-expressing or CD69-expressing CD4^+^ T cells (B); Intensity of CD25 or CD69 expression (C). CFSE-labeled OT-II CD4^+^ T cells were cultured alone or with BMDCs or CT-BMDCs pulsed with the indicated concentration of OVA_323-339_ peptide. At day 4, CFSE dilution was assessed in OT-II CD4^+^ T cells (D). At day 5, IL-2 was detected in the co-culture of OT-II CD4^+^ T cells with BMDCs or CT-BMDCs by cytokine bead assay (eBioscience) (E). ***p<0.001 (Student’s *t*-test). Histograms and data are from one experiment representative of two independent experiments. Data are average ± SEM of triplicate wells.

To examine whether CT modulates the APC capability to activate and proliferate CD4 T cells, we analyzed the expression of activation markers such as CD25 and CD69, IL-2 production and proliferation of responding cells following co-culture of CD4^+^ T cells and CT-BMDCs. CD25 [18] and CD69 [19] are conventional activation markers that are promptly increased following TCR stimulation, and their levels are known to be proportional to strength of TCR signaling [20, 21]. OT-II CD4^+^ T cells were stimulated with BMDCs or CT-BMDCs treated with variable concentrations of peptide and analyzed for expression of CD25 and CD69 at 16 h. As shown in Fig 3A and 3B, the frequency of OT-II CD4^+^ T cells expressing CD25 and CD69 and intensity of the activation markers increased depending on the concentration of pulsing peptide, respectively. However, CT-BMDCs-stimulated OT-II CD4^+^ T cells showed significantly reduced levels of CD25 and CD69 compared to the cells stimulated with BMDCs (Fig 3C).

We also compared proliferation of OT-II CD4^+^ T cells stimulated with BMDCs and CT-BMDCs. CFSE-labeled OT-II CD4^+^ T cells were cultured with BMDCs or CT-BMDCs, and after 4 days, the CFSE dilution was assessed for OT-II CD4^+^ T cells. As shown in Fig 3D, the proliferation of responding cells was enhanced proportionally to pulsing peptide in both co-cultures containing BMDCs and CT-BMDCs, but CT-BMDC-stimulated OT-II CD4^+^ T cells showed significantly reduced proliferation with all concentrations of pulsing peptide. IL-2 production was also reduced in co-culture with CT-BMDCs compared to BMDC-containing co-culture (Fig 3E). Taken together, these results suggest that CT can modulate the stimulating potential of BMDCs to responding CD4^+^ T cells.

### CT directs BMDCs to polarize Th17 cell differentiation by modulating the strength of TCR signaling

**Fig 4:**
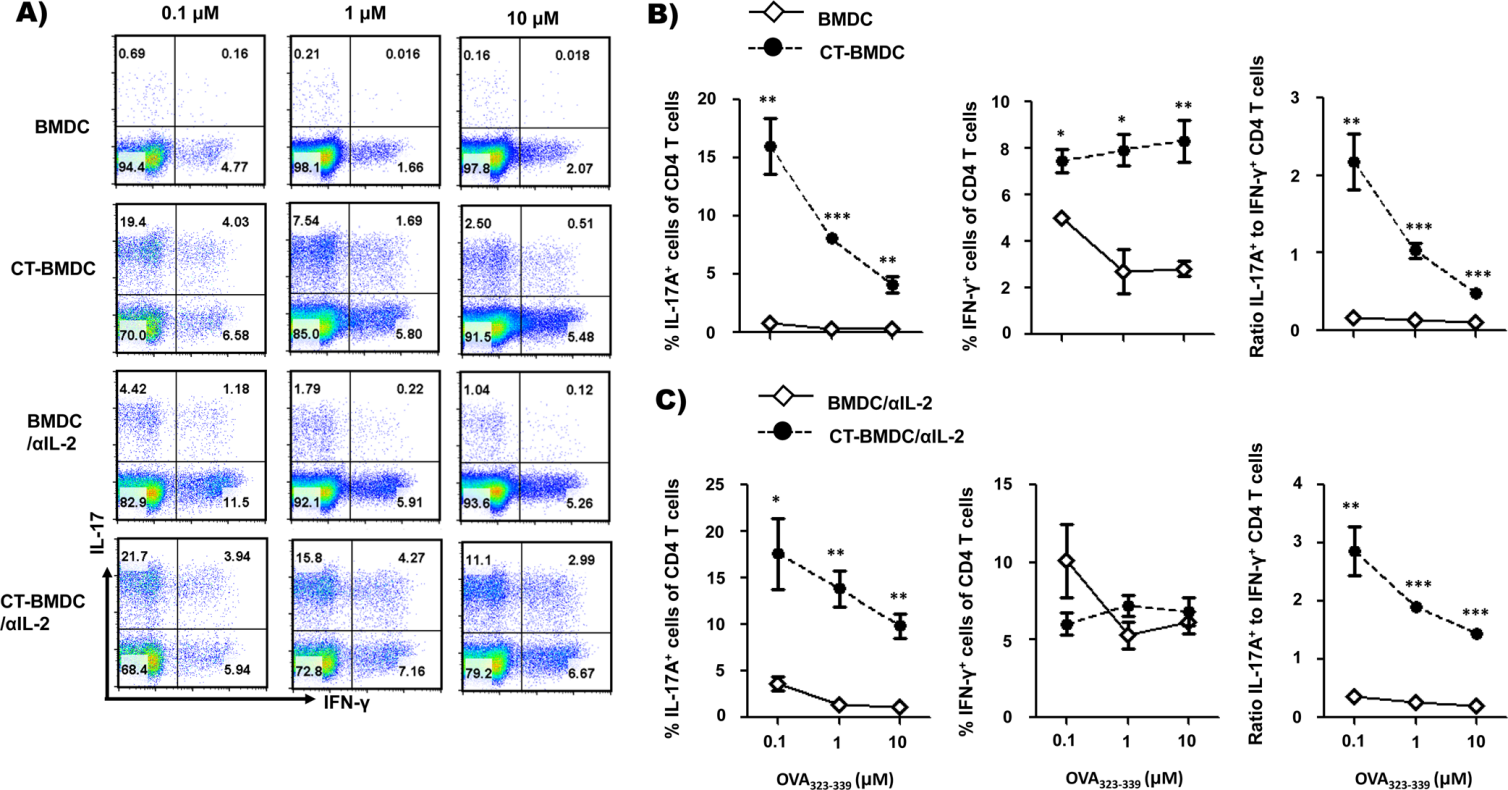
Depletion of IL-2 recovers Th17 cell differentiation. Assessment of OT-II CD4^+^ T cell differentiation by untreated or CT-treated BMDCs pulsed with the indicated concentration of OVA_323-339_ peptide in the absence (A and B) or presence of neutralizing IL-2 antibody (10 μg/ml) (A and C). *p<0.05, **p<0.01, ***p<0.001 (Student’s *t*-test). Dot plots and data are from one experiment representative of at least two independent experiments with similar results and average ± SEM of triplicate wells.

To determine whether CT influences TCR signaling in BMDC-mediated Th cell differentiation, we tested Th17 cell differentiation using BMDCs or CT-BMDCs pulsed with various concentrations of OVA peptide. To this end, BMDCs were treated or not treated with CT for 24 h, pulsed with 0.1 μM to 10 μM concentrations of OVA_323-339_ peptide for 1 h and co-cultured with OT-II CD4^+^ T cells for 5 days. As shown in Fig 4A and 4B, the frequency of IL-17A-producing cells was decreased with increasing concentrations of the peptide in both co-cultures but fairly increased in CT-BMDC-containing co-cultures compared to BMDC-containing co-cultures. In contrast, the frequency of IFN-γ-producing cells was comparable in CT-BMDC-containing co-cultures, independently of the concentrations of pulsing peptide and decreased in BMDC-containing co-cultures with increasing concentrations of the peptide (Fig 4A and 4B).

Previously, we found that IL-2 was increased proportionally to the concentration of pulsing peptide both in BMDC- and CT-BMDC-containing co-culture supernatant, but CT-BMDCs induced relatively low levels of IL-2 in comparison to BMDCs (Fig 3E). Because IL-2 signaling was shown to inhibit Th17 cell differentiation [22–24], we hypothesized that CT supports Th17 cell differentiation by regulating IL-2 production through modulation of the stimulating potential of BMDCs. Therefore, we tested Th17 cell differentiation in the absence or presence of a neutralizing antibody to IL-2. Indeed, Th17 cell differentiation by CT-BMDCs was significantly increased even with 10 μM peptide stimulation when the co-culture was treated with anti-IL-2 neutralizing antibody (Fig 4A–4C). In the presence of neutralizing IL-2 antibody, CT-BMDCs recovered the skewed Th17 cell differentiation even at high concentrations of pulsing peptide (1 μM and 10 μM), in which the skewed Th17 cell differentiation was not supported (Fig 4A and 4C). Taken together, these results suggest that CT modulate BMDCs to favor Th17 differentiation by decreasing TCR signaling and by reducing IL-2 production of responding CD4^+^ T cells, which inhibits Th17 cell differentiation.

### CT induces inflammatory cytokines including activin A in BMDCs

**Fig 5:**
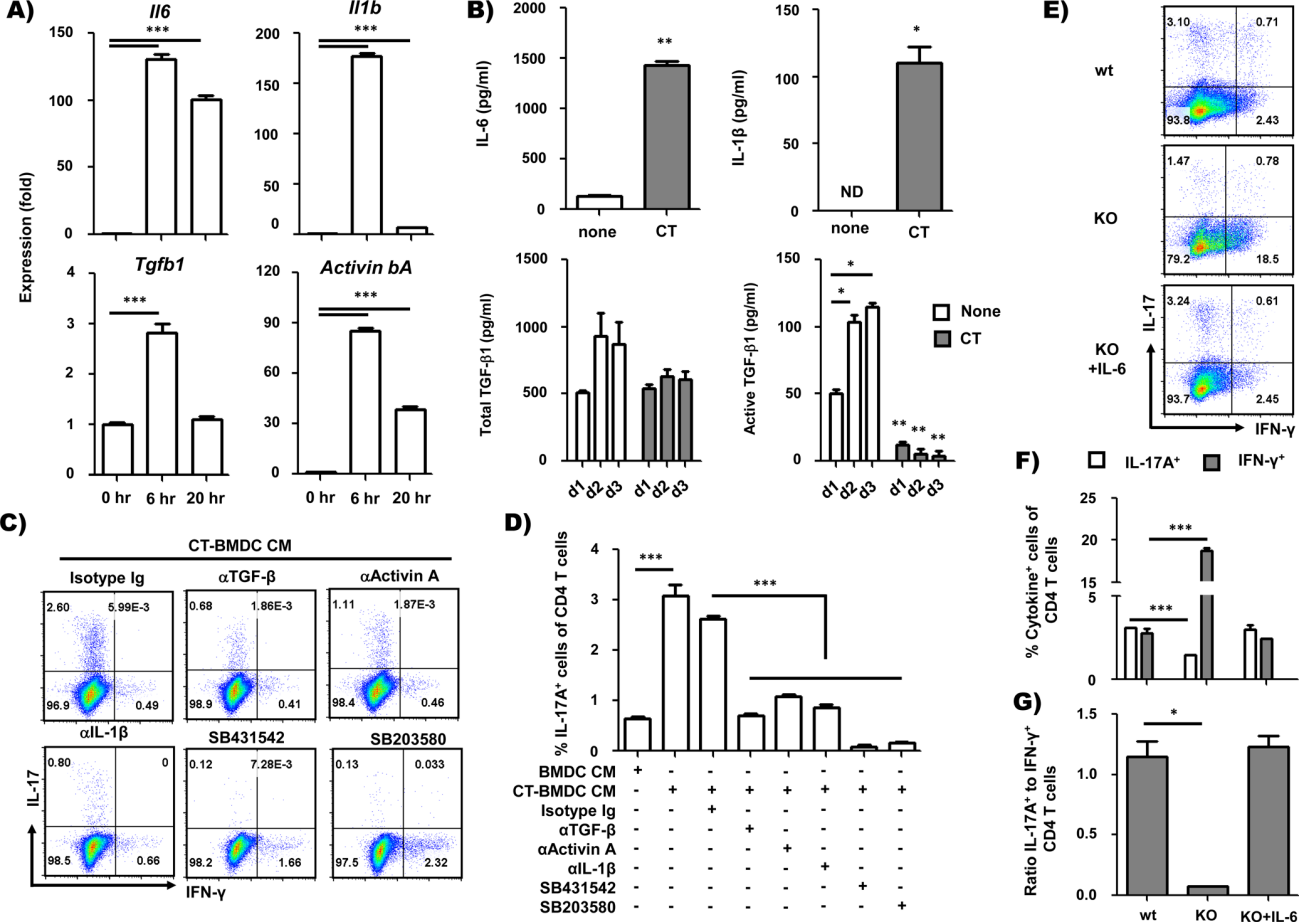
CT–treated BMDCs mediate Th17 cell differentiation by producing Th17 polarizing cytokines. (A) RT-PCR analysis of IL-6, IL-1β, TGF-β1 and activin A mRNA in BMDCs. BMDCs were cultured with CT (2 μg/ml) and harvested at 0, 6, or 20 h after CT treatment. mRNA levels of IL-6, IL-1β, TGF-β1 and activin βA were normalized by mRNA expression of GAPDH and β-actin. (B) Determination of cytokines in BMDC-conditioned media. BMDCs were cultured with CT (2 μg/ml) for 2 days, and culture media was removed to measure cytokines. IL-6 and IL-1β were assayed by multiplex bead cytokine assay kit following the manufacturer’s recommended protocol (eBioscience). TGF-β1 in BMDC-conditioned media was assayed for total (left) and active form (right) by ELISA (R&D Systems). (C and D) Intracellular staining of IFN-γ and IL-17A in OT-II CD4^+^ T cells stimulated with anti-CD3 and anti-CD28 antibody in the presence of BMDC-CM or CT-BMDC-CM for 5 days and restimulated with PMA and ionomycin after 5 days. Neutralizing antibodies and kinase inhibitors were also added in the culture as indicated. (E-G) BMDCs from *IL-6*^*-/-*^ mice were used for clarifying a role of IL-6 in Th17 differentiation promoted by CT-treated BMDCs. Frequency of IFN-γ^+^ or IL-17A^+^ CD4^+^ T cells (F) and ratio of IL-17^+^ cells to IFN-γ^+^ cells (G). *p<0.5, **p<0.01, ***p<0.001 (Student’s *t*-test). Data are the representative of at least two independent experiments with similar results and average ± SEM of triplicate wells in A and D or duplicate wells in B, F and G.

Previously, we found that CT-mediated Th17 cell differentiation was significantly enhanced by depleting IL-2 in high concentrations (1 μM and 10 μM; *p* = 0.04 and *p* = 0.02, respectively, Student’s *t*-test) of peptide, but not in low concentrations (0.1 μM; *p* = 0.74, Student’s *t*-test) of peptide (Fig 4B and 4C). As shown in Fig 3E, in low concentration (0.1 μM) of peptide, the level of IL-2 was comparable in BMDC and CT-BMDC co-culture but CT-BMDCs still induced significant Th17 cell differentiation compared to BMDCs (Fig 4A and 4B). Thus, it is likely that there are some other factors acting on CT-mediated Th17 cell differentiation in addition to modulating TCR signaling intensity. We examined the soluble mediators of Th17 cell differentiation that were induced by CT treatment in BMDC culture. We first tested the expression of various cytokine genes at 0, 6, and 20 h after CT treatment by performing real-time PCR (RT-PCR). The levels of IL-6 and IL-1β mRNA increased dramatically at 6 h after CT treatment among inflammatory cytokines critical for *in vitro* Th17 differentiation [8, 9]. While the level of IL-1β mRNA decreased at 20 h after CT treatment, the level of IL-6 mRNA was sustained until 20 h (Fig 5A). The level of TGF-β1 mRNA transiently increased at 6 h after CT treatment (Fig 5A). TGF-β2 and TGF-β3 mRNA were not detected at all time points (data not shown).

Previously, it has been shown that activin A, a member of TGF-β superfamily, is capable of driving *in vitro* Th17 cell differentiation with IL-6, replacing TGF-β [25]. Thus, we also analyzed mRNA encoding the activin βA and βB subunit for activin A (βA-βA), activin AB (βA-βB) and activin B (βB-βB) [26]. The level of activin βA mRNA was dramatically increased at 6 h and subsequently declined at 20 h after CT treatment, similar to IL-1β mRNA (Fig 5A). In contrast, activin βB mRNA was not detected at any time point (data not shown). The *nodal* and *activin α* subunit, another member of the TGF-β family, and the α subunit of inhibin, which inhibits activin A by competing for activin receptors, were also analyzed but not detected at all time points (data not shown). We also confirmed the production of these cytokines in BMDC cultures incubated with CT for 48 h. As shown in Fig 5B, IL-6 and IL-1β were increased in CT-BMDCs, as reported in the previous studies [8, 9]. We also analyzed total and active TGF-β1 by ELISA assay, and both total and active TGF-β1 were reduced significantly in the culture supernatant of CT-treated BMDCs. Other inflammatory cytokines such as MCP-1, MCP-3, IL-10, IL-18, IL-12p70, IL-23, TNF-α, MIP-1α and MIP-1β were also analyzed in the culture supernatant of CT-treated BMDCs, but none showed significant differences. These data indicate that CT induces Th17-driving cytokines such as IL-1β, IL-6, and activin A in BMDCs.

### Activin A has a critical role in Th17 differentiation by CT *in vitro*

We next examined whether the cytokines produced by CT-treated BMDCs are necessary for Th17 cell differentiation. Naive CD4^+^ T cells were stimulated with plate-coated anti-CD3 antibody and soluble anti-CD28 antibody in the presence of untreated BMDC-conditioned media (CM) or CT-treated BMDC-CM. Neutralizing antibodies to TGF-β1, activin A and IL-1β were included during the culture period to determine the effect of each cytokine on Th17 cell differentiation by CT. Compared to the isotype control, the addition of each neutralizing antibody resulted in reduced frequencies of IL-17-producing T cells (Fig 5C). Although TGFβ1 and activin A bind different receptors, they have the same signaling pathway. Interestingly, neutralization of either cytokine inhibited Th17 cell differentiation by CT-treated BMDC-CM, suggesting that TGF-β1 and activin A act synergistically on Th17 differentiation by CT.

The effect of the TGF-β signaling pathway in Th17 cell differentiation by CT-treated BMDC-CM was further confirmed by using inhibitors of the TGF-β signaling pathway. SB431542 is a well-known inhibitor of the type I receptor kinases for TGF-β family members. SB431542 inhibited Th17 cell differentiation almost completely (Fig 5C and 5D). SB203580, an inhibitor of p38 MAPK, a member of the TGF-β signaling pathway, also inhibited Th17 cell differentiation by CT-treated BMDC-CM (Fig 5C and 5D). Neutralization both of TGF-β and activin A inhibited Th17 cell differentiation by CT-BMDC CM comparably to SB431542 (S3 Fig). This result reveals that activin A, together with TGF-β1, has a critical role in Th17 cell differentiation by CT.

We also examined the role of IL-6 in Th17 differentiation by using *IL-6*^*-/-*^ BMDCs. As shown in Fig 5E–5G, CT-treated *IL-6*^*-/-*^ BMDCs were incapable of inducing Th17 differentiation, and the addition of exogenous IL-6 restored skewed Th17 cell differentiation in the culture.

## Discussion

In this study, we aimed to investigate the mechanisms involved in preferential Th17 differentiation by CT administration. In experimental systems involving *in vivo* immunization and *ex vivo* Th cell differentiation, we determined which DC subset has a critical role in Th17 differentiation by CT administration. Intranasal CT administration primes DCs to migrate to the lung draining lymph nodes, and the CD103^+^ and CD11b^hi^ migratory DC subsets are comparably recruited to the medLN. Our *ex vivo* study shows that of those DC subsets, CD11b^hi^ DCs have a major role in Th17 polarization by CT administration. Consistent with these results, *Batf3*^*-/-*^ mice, which are deficient in CD103^+^ DCs [27], showed enhanced Th17 response after CT treatment, similar to wild type littermates, suggesting that CD103^+^ DC subsets are not essential for Th17 differentiation by CT administration (data not shown). Other group reported that CT promotes Th17 response by acting on CD11b^+^ DCs [28]. It has also been shown that IRF4-dependent CD11b^+^ DCs control mucosal Th17 responses both in human and mouse [29, 30], further supporting our results that the CD11b^hi^ DC subset is specialized in driving Th17 responses.

We found that each DC subset displays a different maturation status. CD103^+^ DC subset displayed relatively matured phenotypes compared to the CD11b^hi^ DC subset, which sustained an immature status after CT administration. Interestingly, *in vitro* CT treatment also resulted in the phenotypic changes in BMDCs as determined by the reduced expression of MHC class II and CD86. Such phenotypic changes led to reduced T cell activation and increased Th17 cell differentiation, which suggests that CT supports Th17-skewed differentiation by modulating the stimulating potential of APCs. Co-culture of naïve CD4^+^ T cells with CT-treated BMDCs exhibited reduced IL-2 production, which is known to suppress Th17 cell differentiation [22, 23]. Thus, our data suggest that CT directs BMDCs to promote Th17 cell differentiation by lowering the strength of the TCR signal and subsequently by reducing IL-2 production. Consistent with our results, it has been shown that low-strength TCR signaling enhances Th17 responses in the presence of anti-CD28 [3], and CD28/B7 co-stimulation negatively regulates Th17 differentiation via IL-2- and IFN-γ-dependent mechanisms [31]. Recently, it has also been reported that TCR-dependent signal intensity takes priority in CD4^+^ T cell polarization *in vivo* to cytokine milieu through dictating expression of the receptor for Th polarizing cytokines [7]. Taken together, our results suggest that the intensity of TCR-dependent and costimulatory signaling is one of the critical factors to control Th17-cell development in addition to polarizing cytokines.

CT also stimulates BMDCs to produce Th17 polarizing cytokines, including activin A. Activins are members of the TGF-β superfamily, dimeric proteins consisting of βA and βB subunits (activin A (βAβA), B (βBβB) and AB (βAβB)) that have variable biological functions in inflammation, hematopoiesis and organogenesis [32, 33]. TGF-β and activin A bind heteromeric receptors consisting of type I and type II receptors and transduce signals through a shared pathway [34]. Our cytokine depletion study showed that activin A has a synergic role with TGF-β1 in CT-directed Th17 cell differentiation. These results are partly consistent with a previous study showing that activin A is capable of inducing *in vitro* Th17 cell differentiation together with IL-6, replacing TGF-β [25].

In conclusion, our study demonstrates that CT induces DCs to promote Th17 cell differentiation by inducing polarizing cytokines and by modulating TCR-dependent and costimulatory signals. Our observations advance the understanding of the cellular and molecular mediators involved in Th17 differentiation by using a Th17-polarizing adjuvant, cholera toxin. This study will aid the development of strategies for modulating the Th17 response by targeting DCs.

## Supporting Information

S1 Fig Phenotypes of MHC II^lo^CD86^lo^ BMDC populations. BMDCs were obtained as in Fig 2E and analyzed for surface expression of MHC class II and CD86 by flow cytometry. (A) Frequencies of MHC II^lo^CD86^lo^ population (square area) and (B and C) mean fluorescence intensity (MFI) for MHC class II and CD86 of the population.
(TIF)

S2 Fig Number of IL-17A- and IFN-γ-producing CD4 T cells induced by mixed, MHC II^lo^CD86^lo^ or MHC II^hi^CD86^hi^ populations either from BMDCs or CT-BMDCs. Data are corresponding to Fig 2F–2I. **p<0.01, ***p<0.001 (Student’s *t*-test). Data are average ± SEM of quadruplicate wells.
(TIF)

S3 Fig TGF-β and activin A have a critical role in CT-mediated Th17 cell differentiation in synergic manner. *In vitro* Th cell differentiation was performed as in Fig 5D. Frequency of IL-17A-producing CD4^+^ T cells. **p<0.01, ***p<0.001, ns = non-significant (Student’s *t*-test). Data are average ± SEM of quadruplicate wells.
(TIF)
