# Supplementary figures and images for: Cholera Toxin Promotes Th17 Cell Differentiation by Modulating Expression of Polarizing Cytokines and the Antigen-Presenting Potential of Dendritic Cells
Source: PLoS One. 2016 Jun 6;11(6):e0157015. doi: 10.1371/journal.pone.0157015 (PMC4894639; doi:10.1371/journal.pone.0157015)

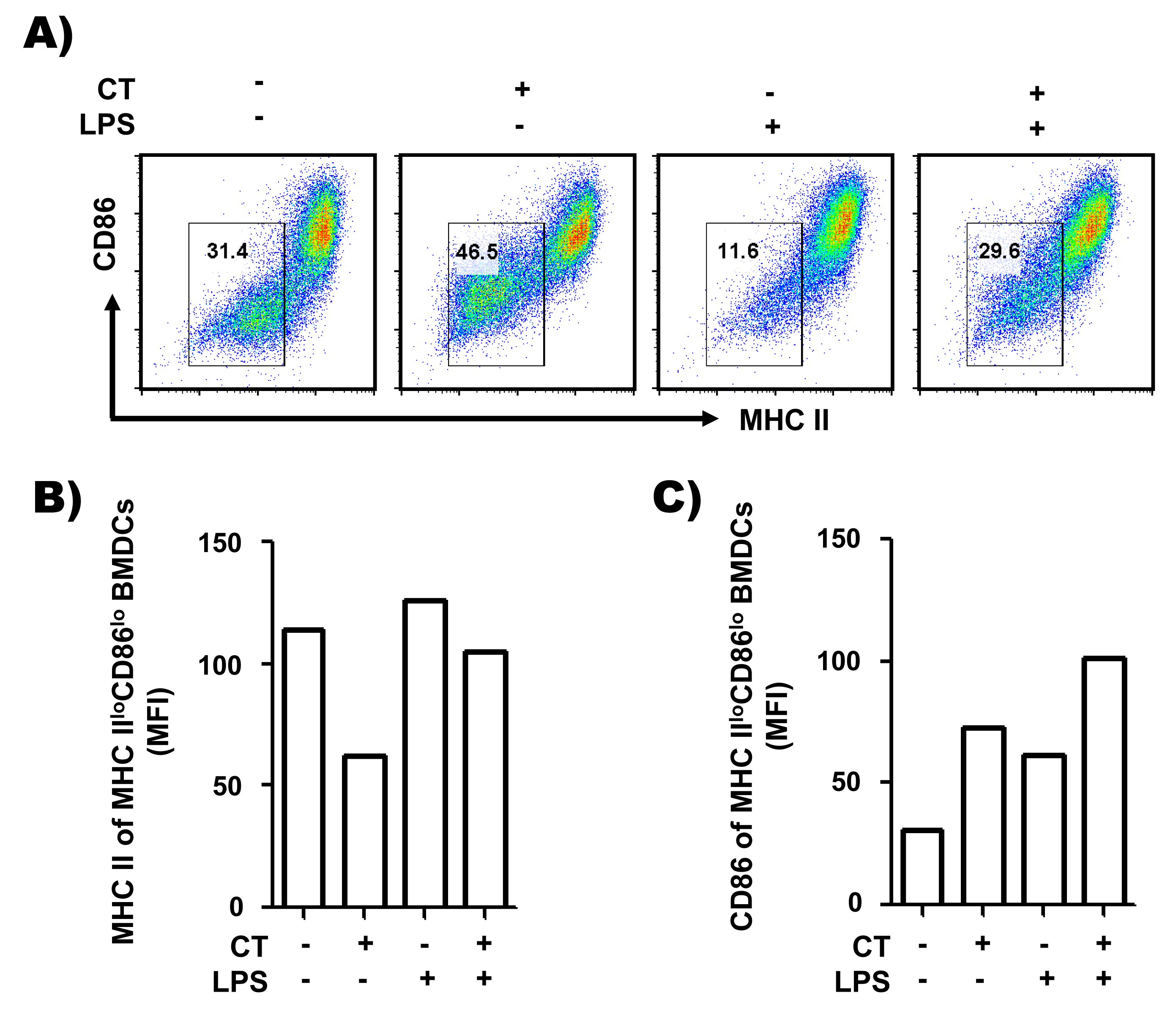

Supplement: S1 Fig — BMDCs were obtained as in Fig 2E and analyzed for surface expression of MHC class II and CD86 by flow cytometry. (A) Frequencies of MHC IIloCD86lo population (square area) and (B and C) mean fluorescence intensity (MFI) for MHC class II and CD86 of the population. (TIF) [file pone.0157015.s001.tif]

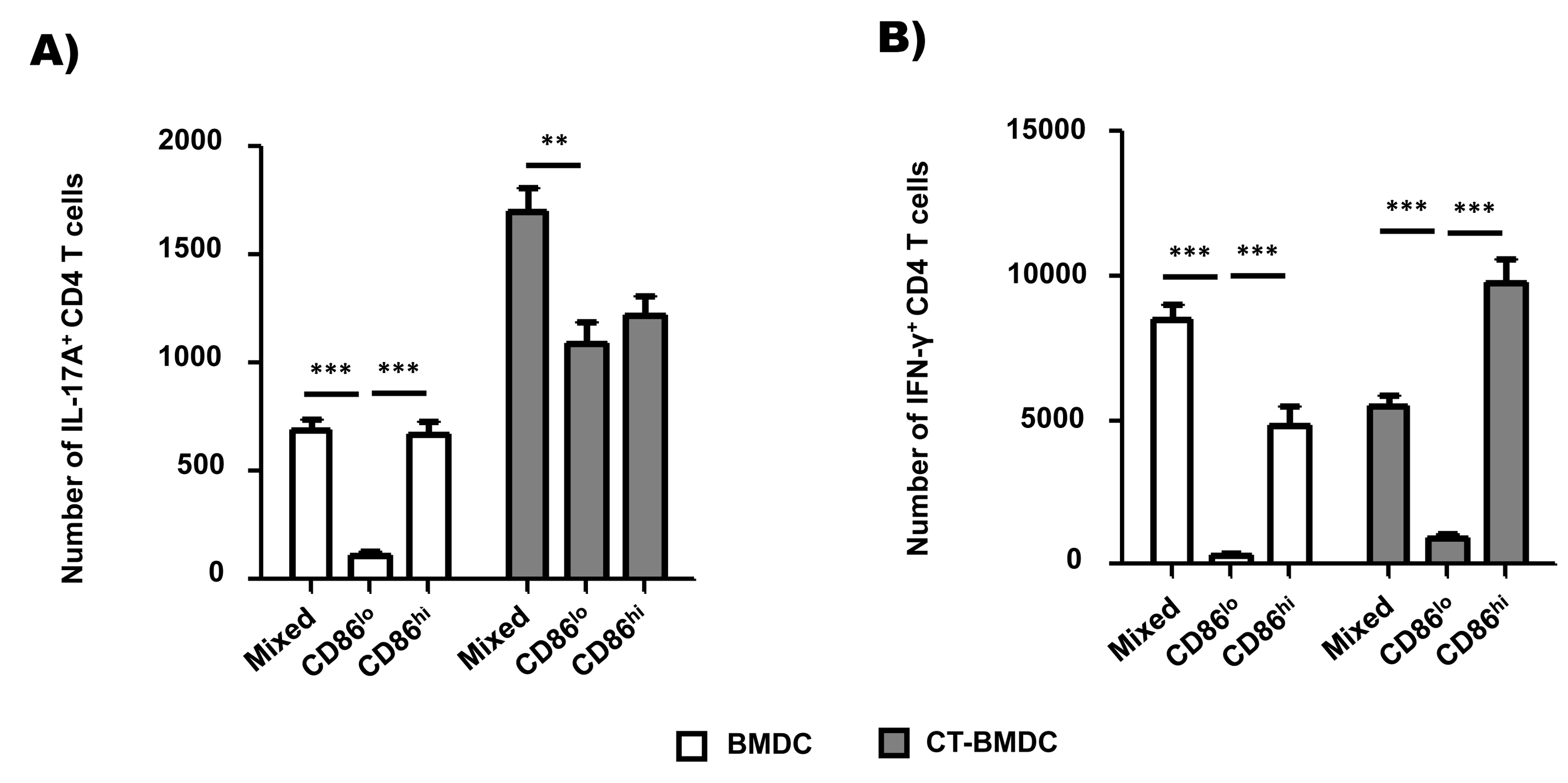

Supplement: S2 Fig — Data are corresponding to Fig 2F–2I. **p<0.01, ***p<0.001 (Student’s t-test). Data are average ± SEM of quadruplicate wells. (TIF) [file pone.0157015.s002.tif]

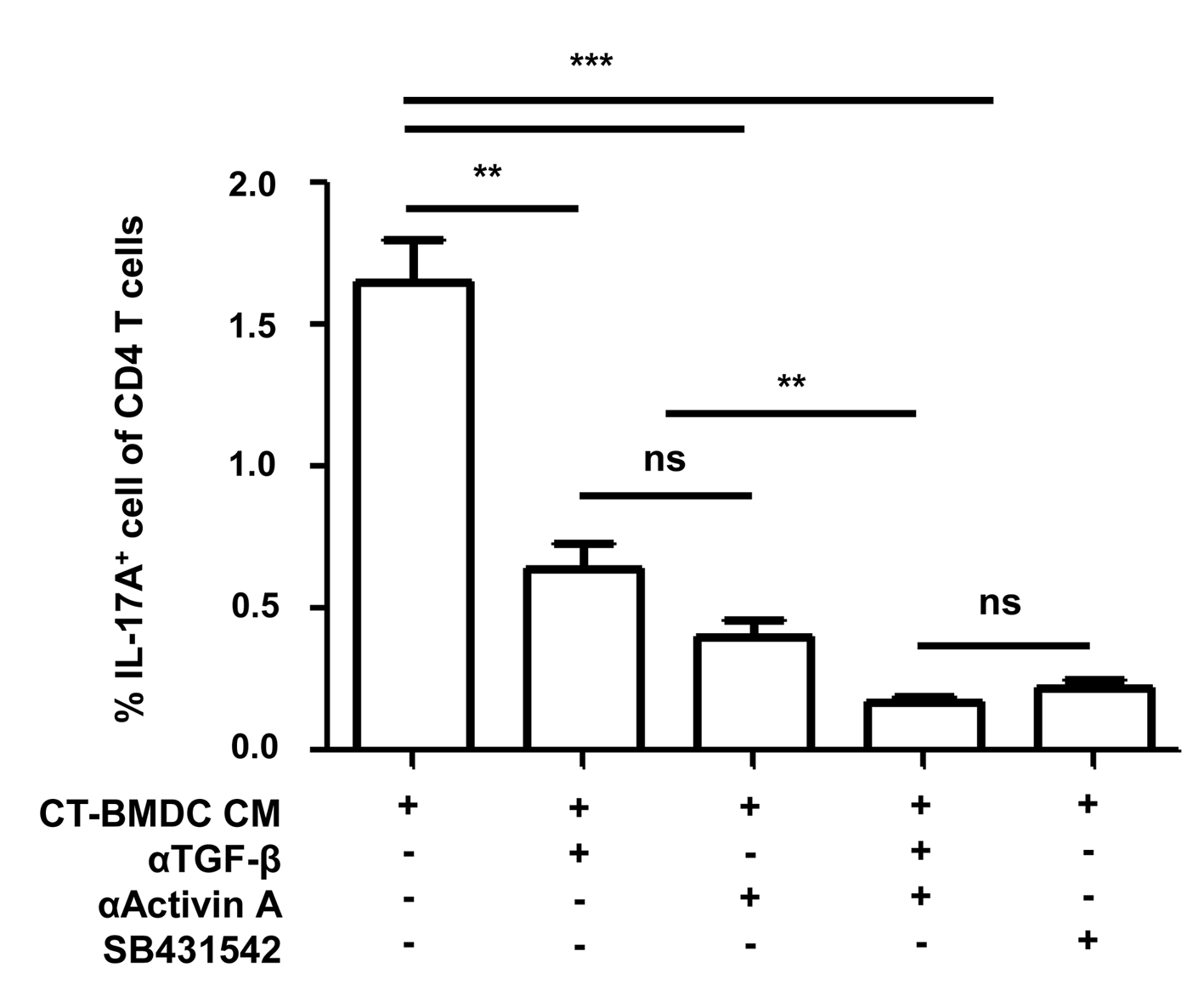

Supplement: S3 Fig — In vitro Th cell differentiation was performed as in Fig 5D. Frequency of IL-17A-producing CD4+ T cells. **p<0.01, ***p<0.001, ns = non-significant (Student’s t-test). Data are average ± SEM of quadruplicate wells. (TIF) [file pone.0157015.s003.tif]
